# Functional disease progression in children with inherited peripheral neuropathies: A prospective cohort study

**DOI:** 10.1177/22143602261450041

**Published:** 2026-07-03

**Authors:** Katharina Vill, Moritz Tacke, Anna König, Jan Senderek, Iris Hannibal, Maggie C. Walter, Beate Schlotter-Weigel, Simone Thiele, Johanna Wagner, Moritz Netzer, Wolfgang Müller-Felber, Elke Hobbiebrunken, Astrid Blaschek

**Affiliations:** 1 Department of Pediatric Neurology and Developmental Medicine and LMU Center for Children with Medical Complexity, Dr. von Hauner Children’s Hospital, LMU Hospital, Ludwig-Maximilians-University, Munich, Germany; 2 Department of Pediatrics and Adolescent Medicine, Division of Pediatric Neurology, University Medical Centre Göttingen, Georg August University Göttingen, Germany; 3 Friedrich Baur Institute at the Department of Neurology, LMU University Hospital, LMU Munich, Munich, Germany

**Keywords:** charcot marie tooth disease, hereditary motor and sensory neuropathy, natural history, outcome measures, childhood CMT

## Abstract

**Objective:**

Charcot-Marie-Tooth disease (CMT) is the most common inherited peripheral neuropathy and usually manifests in the first two decades of life. However, prospective data on the natural course during childhood remain scarce.

**Methods:**

This two-year, prospective, observational cohort study was conducted at two German pediatric neuromuscular centers. Annual standardized clinical assessments were performed. Sixty-eight pediatric patients with CMT (age range: 4–18 years; 35 females) were included. The primary outcome was the CMTPedS score, assessed at baseline and after a mean interval of 2.03 ± 0.23 years. Changes in the 11 individual CMTPedS subitems were also analyzed.

**Results:**

The mean baseline CMTPedS score for the total cohort (n=68) was 20.5 ± 8.8. Stratified by genotype, mean scores were: CMT1A (n=31), 16.5 ± 7.6; CMT1B (n=6), 21.5 ± 9.4; CMT2A (n=4), 35.8 ± 10.0; CMT4C (n=4), 27.0 ± 3.7; CMTX (n=4), 24.3 ± 10.1); others (n=13), 24.6 ± 7.7; genetically unsolved (n=6), 19.8 ± 8.6. Over a two-year observation period, the mean change in CMTPedS was 0.0 ± 2.7, indicating overall no significant progression.

**Conclusion:**

In this well-characterized cohort, overall disease course was non-progressive over two years—most notably in CMT1A, the most prevalent subtype. CMT2A and CMT1B showed numerically greater changes, yet none reached statistical significance. These results emphasize the slow-progressive nature of pediatric CMT and highlight the limitations of using short-term clinical progression as an outcome measure in therapeutic trials.

## What is already known on this topic

A similar study was published by Cornett et al. in 2017. Two hundred and six participants aged 3–20 years were assessed at baseline and after two years. Using the CMTPedS to measure disease severity, the researchers found that children with CMT showed an average disease progression of 2.4 ± 4.9 points over two years, which was considered statistically significant (p < 0.001).

## What this study adds

We are increasing the number of patients followed longitudinally with the CMTPedScale by additional 68 patients. We provide annual measurements of the score and can estimate the disease progression for five CMT subtypes per year. Interestingly, our CMT1A cohort did not show a significant worsening over the observation period. These findings are statistically compatible, however, suggesting that our study captures a subset of patients at the stable end of the disease spectrum and that younger children and a milder baseline might mask the detection of significant progression in such a slowly evolving disease.

## How this study might affect research, practice or policy

Natural history studies can help establishing valid and responsive outcome measures for clinical research, including interventional studies testing new treatments. Based on our data, it appears that in interventional trials involving pediatric CMT patients, the absence of deterioration over a 2–3-year period may not necessarily indicate treatment efficacy, but rather reflect the natural course of the disease.

## Introduction

Hereditary motor and sensory neuropathies (synonym: Charcot-Marie-Tooth disease, CMT) are a group of monogenic neurogenetic disorders caused by mutations in more than 80 different genes. They are among the most common inherited neurological disorders with a prevalence of 20-30 per 100,000 individuals.^1^

Although the phenotypic presentation varies,^2^ most patients present in the first or second decade of life with distal weakness, paraesthesia and foot deformities. The disease usually progresses slowly over the entire lifespan, although some patients may develop severe, rapidly progressive disability,^3,4^ and there may occur dramatic, acute symptoms in exceptional cases.^5^

CMT has historically been divided into “demyelinating forms” and “axonal forms” based on neurophysiological and neuropathological features. With increasing knowledge of the underlying genetic defects, the nomenclature has expanded into a complex list of CMT subtypes. Autosomal-dominantly inherited demyelinating CMT1A is the most common form caused by duplication of the *PMP22* gene.

Currently, there is no curative treatment for CMT; however, innovative therapeutic approaches are now being evaluated in clinical trials.^6^ A major challenge in assessing the efficacy of new therapies, especially in pediatric patients, is the lack of reliable outcome measures, largely due to the slow progression of most CMT subtypes and limited natural history data.

To address this gap, Fridman and colleagues performed a large cross-sectional analysis including 1652 CMT patients, of whom 423 were children and adolescents under 21 years of age.^4^ However, all patients were assessed using the CMT Neuropathy Score (CMTNS), a tool validated for adults, and outcomes specific to the pediatric cohort were not explicitly reported.

In 2012, Burns et al.^7^ validated the Charcot-Marie-Tooth disease pediatric scale as an outcome measure of disability. Children aged 3 to 20 with various types of Charcot-Marie-Tooth disease (CMT) were recruited across multiple countries. Through rigorous development stages including item generation, peer review, and pilot testing involving 172 patients, the CMT Pediatric Scale (CMTPedS) comprising 11 items across seven domains (strength, dexterity, sensation, gait, balance, power, endurance) was validated. Rasch analysis confirmed its reliability, unidimensionality, and sensitivity as a disability measure for children with CMT, completing in 25 minutes and applicable from age 3 upwards. In 2017, Cornett et al. assessed 206 children with CMT at baseline and after 2 years, using the CMTPedS, and found that CMT progressed at a significant rate in pediatric patients.^8^

The primary aim of this study was to document disease progression of childhood CMT over a 2-year period. In addition, our study focused on investigating whether different CMT subtypes exhibit differences in disease severity.

## Patients and methods

A cohort of 68 children and adolescents (33 males, 35 females) with different subtypes of isolated, non-syndromic hereditary motor and sensory neuropathy CMT could be included into the study. 43 patients were under regular medical care at our Paediatric Neuromuscular Centre at the Dr. v. Hauner Children’s Hospital, Ludwig-Maximilians-University, Munich, Germany, and 25 patients were under regular medical care at the Department of Paediatric Neurology, University of Göttingen, Germany. Inclusion criteria were a clinical and neurophysiological diagnosis of CMT, age 4-18 years, data on baseline, a 2-year study visit (±6 months) and written informed consent.

This was a two-year, prospective, observational cohort study. Using the CMTPedS, patients were recorded and evaluated annually.^9^ Data collection was performed between 06/2018 and 06/2022. Personal and clinical information (age, sex, age at onset, symptoms, neurophysiology, complications, use of assistive devices and genetic information, and baseline CMTPedS) can be found in the Supplementary Table.

CMTPedS items included hand dynamometry for grip, dorsiflexion, and plantarflexion strength, and pinprick and vibration testing for sensory assessment. Balance, gait, long jump and 6-minute walk test were further items. The balance and gait items were themselves composed of subtests; balance was administered using the Bruininks-Oseretsky Test of Motor Proficiency, 2nd Ed (BOT-2). The tests were carried out using the “Charcot-Marie-Tooth disease Pediatric Scale equipment and training resource kit” by physiotherapists with many years of experience in clinical trials in the field of neuromuscular disorders. To obtain z-scores, raw scores were converted to age- and sex-matched normative reference values from the 1000 Norms Project by the respective authors.^7,8,10^ A score of 1, 2, or 3 represents 1 to 2SD, 2 to 3SD, or 3 to 4SD below normal, and 4 represents >4SD below normal. A CMTPedS score of 0–14 is considered mild, 15–29 moderate, and 30–44 severe.^8^ All tests took place on a single day in the respective outpatient clinics and were performed as part of the routine outpatient follow-up of CMT patients. Standardised conditions were ensured by using the same sequence and sufficient resting time between each session. Paired sample Wilcoxon rank test was used to measure statistical levels of significance.

## Results

### Study population and distribution of CMT subtypes

**Figure 1.**
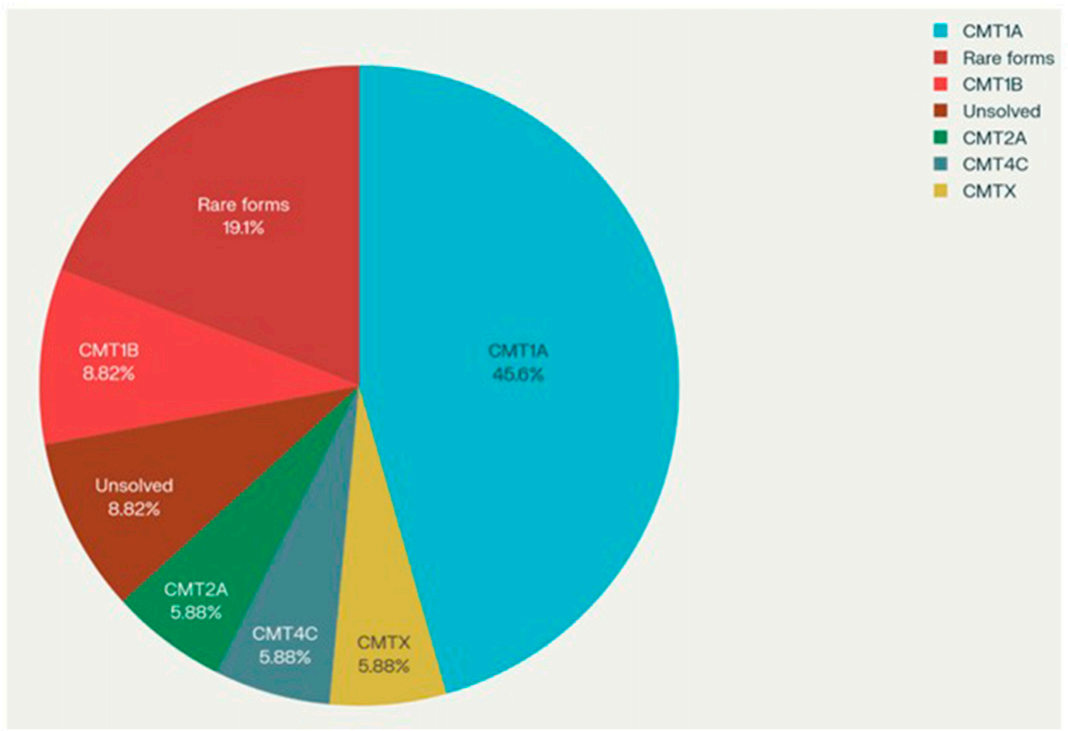
Frequency of genetic CMT subtypes in the cohort (n=68).

Natural history data were collected at baseline and after two years from 68 children with CMT (35 females; 51.5%), aged 4–18 years (mean age: 11.8 years; median: 12 years). The mean follow-up period was 2.03 ± 0.23 years. Among the 17 molecularly defined CMT subtypes identified in the cohort (see Figure 1: Frequency of genetic subtypes in the cohort), CMT1A (*PMP22* duplication; n = 31, 45.6%) was the most prevalent, followed by CMT1B (*MPZ* mutations; n = 6, 8.8%), CMTX (*GJB1* mutations; n = 4, 5.9%), CMT4C (*SH3TC2* mutations; n = 4, 5.9%), and CMT2A (*MFN2* mutations; n = 4, 5.9%). Six patients (8.8%) remained genetically unclassified despite comprehensive high-throughput sequencing and advanced molecular diagnostics. 13 patients comprised 8 different rare subtypes, occurring only once or twice, including CMT1C (*LITAF* mutation), CMT1E (*PMP22* point mutation), CMT2 (*GDAP1* mutation), CMT2B1 (*LMNA* mutation), CMT4D (*NDRG1* mutation), CMT4F (*PRX* mutation), dominant intermediate CMT E (*INF2* mutation), dominant intermediate CMT F (*GNB4* mutation), Dejerine-Sottas syndrome (*PMP22* point mutation).

Detailed genetic characteristics and the classification criteria for the CMT subtypes identified in our cohort are summarized in Supplementary Table 3, while Refs 11 and 12 provide additional specific information regarding the molecular diagnostics for the rarer genetic variants (*SBF2* and *SACS*) encountered.

#### Level of disability

**Figure 2.**
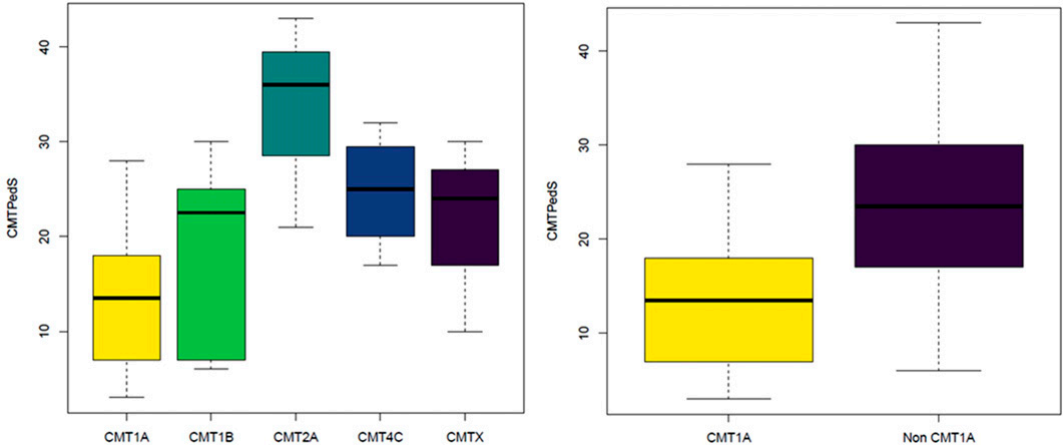
Disease severity of pediatric CMT at baseline assessed using CMTPedS scores. Box plots with median, 2nd/3rd quartiles and range.

At baseline, the mean CMTPedS score for the 68 children with various types of CMT was 20.5± 8.8. Subgroup analyses were conducted for the most common subtype (CMT1A) and for all non-CMT1A cases combined, as well as for the next most frequent subtypes: CMT1B, CMTX, CMT2A and CMT4C (see Figure 2). Children with CMT1A had a median baseline CMTPedS score of 13 (range 3–28), classifying them as ‘mildly affected’; none reached the threshold for ‘severely affected’. The CMT2A cohort displayed the highest severity (median 39.5, range 21–44), with most, but not all, cases classified as ‘severely affected’. Patients with CMT1B (median 24, range 6–33), CMTX (median 27.5, range 10–32) and CMT4C (median 27, range 17–32) had median scores consistent with ‘moderate disease severity’. Notably, CMT4C scores had the narrowest range, with none of the patients being classified as ‘mildly affected’. Baseline severity differed significantly between CMT1A and all other subtypes, as well as between CMT2A and the remaining subgroups (see Figure 2).

#### Intraindividual course of the disease during the observation period

**Figure 3.**
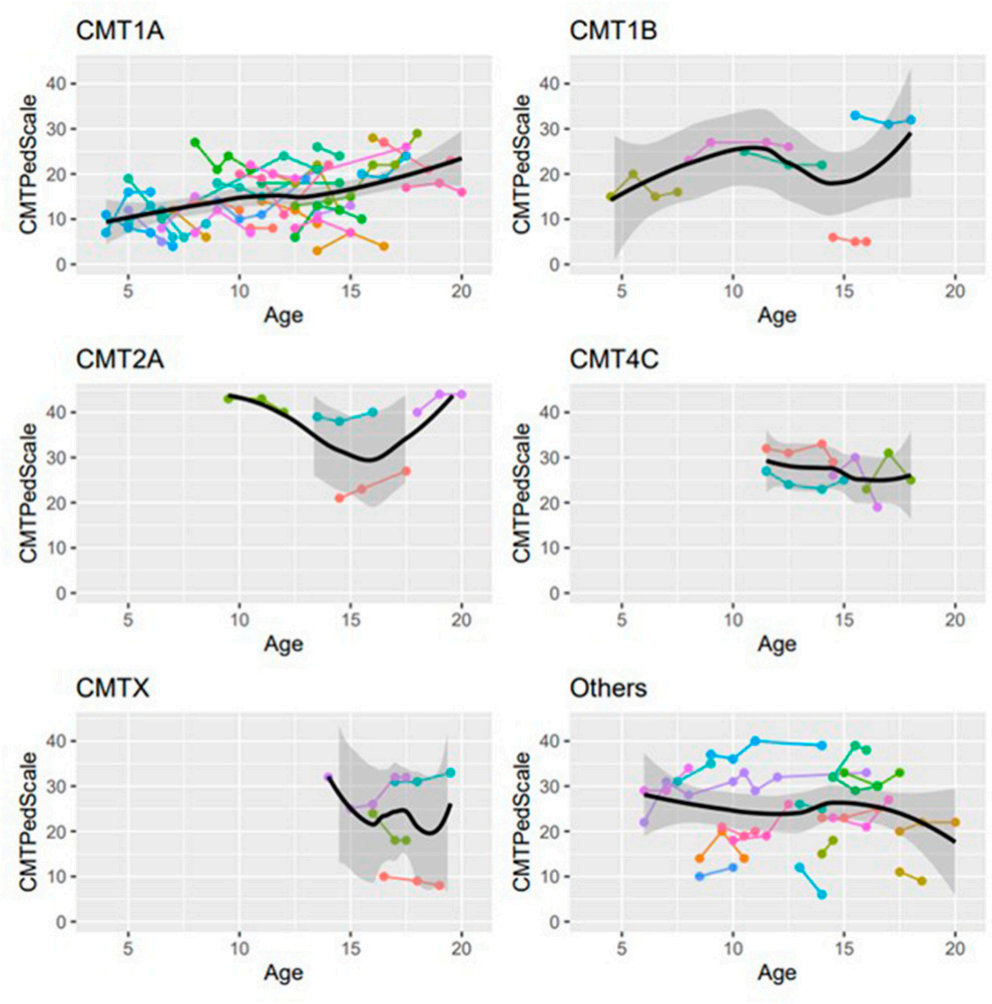
Longitudinal trajectories of individual CMTPedS scores by CMT subtype. Each line represents an individual patient’s score over the observation period, illustrating intraindividual change and stability. The shaded area in each panel represents the distribution of scores within the respective CMT subtype at each time point (e.g., interquartile range or standard deviation), providing a visual summary of group variability and spread of values.

Mean CMTPedS scores for the 68 children with all types of CMT increased by 0.0 ± 2.7 points over the observation period; however, this change was not statistically significant (Table 1; Figure 3).

**Table 1.** Change in CMTPedS total score from baseline to last visit by CMT subtype.

CMT Subtype	n	Baseline Score (mean ± SD)	Last Visit Score (mean ± SD)	Mean Change (mean ± SD)	% Change from Baseline (mean)	Mean Follow-up (years)	p-value
**CMT1A**	31	16.5 ± 7.6	16.6 ± 8.1	0.0 ± 2.7	0.0%	2.2	0.865
**CMT1B**	6	21.5 ± 9.4	21.3 ± 8.0	-0.2 ± 2.3	-0.9%	2.1	1.000
**CMT2A**	4	35.8 ± 10.0	37.8 ± 7.0	+2.0 ± 3.7	+5.6%	2.5	0.375
**CMT4C**	4	27.0 ± 3.7	27.3 ± 2.6	+0.3 ± 3.1	+0.9%	2.6	1.000
**CMTX**	4	24.3 ± 10.1	22.8 ± 11.5	-1.5 ± 3.5	-6.2%	2.4	0.500
**Rare forms**	13	24.6 ± 7.7	28.4 ± 8.5	+2.9 ± 4.1	+11.8%	2.8	0.063
**Unsolved**	6	19.8 ± 8.6	20.0 ± 9.1	+0.2 ± 2.5	+0.8%	1.8	1.000
**Total cohort**	68	20.5 ± 8.8	20.6 ± 9.6	0.0 ± 2.7	0.0%	2.0	0.976

Disease progression is shown according to age, sex, baseline severity and genetic subtype.

Participants were stratified into age groups for the analyses according to Cornett et al. (ages 3–10 and 11–20 years). Disease severity was classified using the established CMTPedS categories of mildly affected (scoring <15 points), moderately affected (scoring 15–29 points), and severely affected (scoring ≥30 points).

Overall, progression rates were similar for both males and females; there was no significant association between age group or baseline CMTPedS score and progression rate. Pairwise comparisons within age groups also revealed no significant differences in CMTPedS change over time.

When stratified by genetic subtype, the 31 participants with CMT1A showed a mean change of +0.0 ± 2.7 points (mean follow-up of 2.2 years), which was not statistically significant (Table 1). Notably, two CMT1A patients demonstrated significant improvement in CMTPedS scores (decreasing from 12 and 6 points, respectively), primarily due to improved sensory performance in pinprick and vibration tests despite examination at all visits by the same, experienced neuropediatrician, and – as were all examining physiotherapists – especially trained for the CMTPedScale. However, excluding these two individuals did not materially affect the statistical outcome. Across the paediatric age span, children with CMT1A progressed at a steady, minimal rate, with no significant differences by sex or influence of baseline age or disease severity on progression.

Of the remaining subtypes, six participants with CMT1B showed a mean change of -0.2 ± 2.3 points (mean follow-up 2.1 years), while those with CMT2A (n = 4) progressed by an average of +2.0 ± 3.7 points (mean follow-up 2.5 years). The CMTX group (n = 4) had a mean change of –1.5 ± 3.5 points, indicating improvement (mean follow-up 2.4years), and the CMT4C group (n = 4) showed a mean change of +0.25 ± 3.1 points (mean follow-up 2.6 years). None of these changes reached statistical significance, and the small sample sizes preclude firm conclusions regarding covariate effects. Additional analysis of sex, age group and baseline severity in these subgroups was limited due to low numbers; the influence of these factors remains inconclusive.

## Sub-item analysis

A detailed assessment of the individual CMTPedS sub-items is tabulated in Table 2 and visualized in Supplementary Figure 4.

**Table 2.** CMTPedS Sub-Item Results at baseline by CMT Subtype (Median [Range]).

Sub-Item	CMT1A (n=31)	CMT1B (n=6)	CMT2A (n=4)	CMT4C (n=4)	CMTX (n=4)	Rare forms (n=13)	Unsolved (n=6)
**Dorsiflexion**	2 [0–4]	4 [1–4]	4 [2–4]	3.5 [2–4]	4 [3–4]	3 [1–4]	2.5 [1–4]
**Plantarflexion**	2 [0–4]	3 [2–4]	4 [3–4]	3 [2–4]	2.5 [2–4]	3 [1–4]	2.5 [1–4]
**Grip Strength**	1 [0–4]	2 [1–3]	4 [0–4]	2 [1–2]	1.5 [1–2]	1 [0–4]	1.5 [0–4]
**9-Hole Peg Test**	0 [0–4]	1.5 [0–4]	4 [1–4]	1 [0–2]	1 [0–4]	2 [0–4]	1.5 [1–3]
**Functional Dexterity**	1 [0–4]	2 [0–4]	4 [0–4]	0.5 [0–1]	2 [0–4]	1 [0–4]	1 [0–4]
**Pinprick**	0 [0–3]	0 [0–0]	1 [0–4]	1.5 [0–4]	0 [0–3]	0 [0–2]	0 [0–2]
**Vibration**	0 [0–3]	2 [0–3]	3 [0–4]	2.5 [0–4]	0 [0–3]	2 [0–3]	1.5 [0–3]
**Balance**	1 [0–4]	3 [0–4]	4 [4–4]	4 [3–4]	2.5 [0–4]	4 [0–4]	2 [0–4]
**6-Minute Walk Test**	1 [0–4]	2 [0–4]	4 [3–4]	2 [1–2]	1.5 [1–4]	2 [1–4]	2 [1–4]
**Long Jump**	2 [0–4]	3 [0–4]	4 [3–4]	3.5 [1–4]	2.5 [0–3]	4 [2–4]	2 [1–4]
**Gait**	2 [0–4]	2 [0–3]	4 [4–4]	2.5 [2–4]	2.5 [2–4]	2 [0–4]	2.5 [0–4]

Distal muscle strength (dorsiflexion, plantarflexion): Pronounced weakness was consistently observed in CMT2A (often near-maximal impairment with low intra-group variability), whereas CMT1A exhibited only mild deficits. CMT4C, CMT1B and CMTX exhibited intermediate values, with CMT4C demonstrating particularly consistent moderate-to-severe weakness.

Grip strength: CMT2A patients had the lowest grip scores, with limited variance. Other subtypes presented a broader range of values, indicating notable individual variability.

Fine motor function (9-Hole Peg Test, functional dexterity): In CMT1A and CMTX, most patients’ scores remained close to the normal range. In CMT2A and CMT4C, however, early and significant impairment was observed. CMT1B exhibited a broad spectrum ranging from normal performance to moderate dysfunction.

Sensory function (pinprick, vibration): Pinprick sensation was strikingly uniform within CMT1B and CMTX, with most individuals showing a consistently preserved irrespective of other functional scores. Sensory impairment in CMT2A varied, with one individual having no problems at all, one intermediate and two with severe deficits – with a tendency of pinprick sensation being less involved than vibration. A wider range of values was as well documented in CMT1A and CMT4C.

Balance: Most CMT2A and CMT4C patients had moderate-to-severe impairment, while CMT1A patients predominantly scored as normal or mildly affected. There was one non-ambulatory CMT2A patient included, and given a balance score of 0.

Endurance and gait (6-minute walk test, long jump): Nearly all CMT2A patients scored at or near maximal severity on the 6MWT, indicating profound limitations in walking capacity. CMT4C also scored poorly, but with slightly more variation. CMT1A, CMT1B and CMTX showed a wider distribution, ranging from near-normal performance to moderate impairment.

These results are presented in tabular and graphic detail in Supplementary Material, including and results for genetically unclassified or rare subgroups.

## Discussion

This prospective, multicenter study provides important new insights into the natural history of pediatric Charcot-Marie-Tooth (CMT) disease, based on a two-year observation-period with annual assessments of 68 children using the Charcot-Marie-Tooth Pediatric Scale (CMTPedS). Our results confirm that childhood CMT progresses so slowly that sensitive tools such as the CMTPedS rarely detect statistically significant clinical worsening over a two-year period. This natural stability poses clear challenges for interventional trials, as treatment effects—particularly those aimed at halting progression—may be difficult to discern against such a background.

The CMTPedS was feasible and well-accepted in the pediatric setting, reliably capturing the outcome parameter of 2-year changes in our cohort. While most patients exhibited minimal progression, these results align with the known gradual degenerative nature of CMT and reinforce the validity of our findings.

A detailed analysis of subitems across CMT subtypes revealed expected genotype-phenotype correlations and informative variability patterns. Distal muscle weakness (dorsiflexion and plantarflexion) was an early and consistent feature. CMT2A patients were the most severely affected across all lower limb domains, demonstrating pronounced gait and endurance limitations (e.g., 6-minute walk test). In contrast, CMT1A patients were typically only mildly impaired in these items. CMT1B, CMT4C, and CMTX patients showed intermediate profiles.

Notably, sensory function (pinprick and vibration) was largely preserved (median 0) in children with CMT1B and CMTX at this age. This contrasts with more severe or adult-onset phenotypes with distinct features in sensory loss.^13,14^

These results suggest that sensory involvement in these subtypes may be mild, progress slowly or become apparent later in life. As expected, higher and more consistent sensory deficits were observed in CMT2A. Balance and fine motor skills were most affected in patients with CMT2A and CMT4C, while patients with CMT1A and CMTX often exhibited milder symptoms, given that the sample sizes are small.

Grip strength and functional dexterity testing exhibited significant heterogeneity within each group. This variability likely reflects the substantial phenotypic spectrum inherent to different CMT subtypes—particularly the notable individual variability seen in CMT1A—as well as the complex interplay between disease progression and normal pediatric maturation, which may influence performance even when using age-normalized z-scores.

The wide distribution of subitem scores in CMT1A is consistent with previous studies that have emphasized substantial phenotypic variability, even within the most common subtype.^2,8,15^ These findings suggest that single sub-items are of limited value as stand-alone markers for disease progression or severity. Instead, a multidimensional evaluation is necessary. By integrating overall disability and domain-specific scores over time, the CMTPedS justifies its use as a robust measurement tool for clinical practice and research.

Importantly, our results demonstrated that disease progression, especially in CMT1A, was negligible over two years. However, the negligible progression observed in our cohort (0.0 ± 2.7 points) falls within the variability reported in larger studies, including the 1-SD range described by Cornett et al., and therefore does not necessarily contradict previous findings. Rather, our results likely reflect the lower end of the expected progression spectrum. A key factor is the mild baseline severity of our CMT1A participants (mean CMTPedS 16.5; median 13), with no patient classified as severely affected. In such mildly affected children, the extremely slow progression characteristic of CMT1A may remain below the sensitivity threshold of clinical rating scales over a 24-month period. Moreover, although the CMTPedS utilizes age- and sex-matched normative reference data to control for maturation, factors like improved task performance through learning and individual developmental gains during childhood can still counterbalance subtle neurodegenerative changes, resulting in apparent clinical stability. In smaller cohorts, this physiological variability and measurement-related ‘noise'—referring to minor, non-pathological fluctuations in test performance or scoring inconsistencies between sessions—may further obscure slow progression and reduce statistical power. Taken together, our findings suggest that short-to medium-term stability can represent a typical disease course in mildly affected pediatric CMT1A and underscore the need for more sensitive outcome measures in clinical trials of limited duration. Surprisingly, the CMTX subtype showed improvement. Given the small number in each group, it was manually checked if factors like undergoing surgery, or participating in an intensive rehabilitation program or testing errors such as wearing ankle-foot orthoses during follow-up testing, might have influenced the results. However, no systemic measurement errors or variability among clinical evaluators could be identified. One must concede that the small sample size, the children’s age when subjective findings such as sensory testing were reported, and the ‘short’ observation period are the problematic factors. Furthermore, as illustrated by the longitudinal trajectories in Figure 3, participants in the CMT2A, CMT4C, and CMTX subgroups were predominantly older at baseline, with data collection beginning primarily in adolescence. This suggests that our study may have captured these patients after their period of most rapid clinical progression toward higher disability scores, potentially contributing to the observed lack of significant change over the two-year window and reinforcing the clinical imperative for early intervention in early childhood.

The intra-individual variability observed in certain sub-items, such as sensory testing and distal muscle strength, warrants consideration. While the CMTPedS utilizes age- and sex-matched z-scores to control for growth, some patients demonstrated notable “jumps” in their intraindividual performance. These fluctuations may be attributed to the subjective nature of sensory testing in children, particularly in younger cohorts. Furthermore, the inherent physiological variability of pediatric performance—where factors like attention, motivation, and ‘daily form’ can impact results—is more likely to influence performance-based items such as balance or endurance tasks. Plus, to maintain a high level of scientific rigor, examiners were instructed not to refer to previous results or to review them before the next visit (proper blinding was of course not possible due to examiner persistence). While this prevents expectation bias, it may also lead to the capture of greater intra-individual ‘noise’ compared to assessments where previous scores are known. This underscores the difficulty of using subjective or effort-dependent pediatric sub-items as isolated biomarkers for progression.

Taken together, our findings highlight the value and limitations of the CMTPedS in pediatric CMT.^9^ It has been used in a variety of clinical and natural history studies since its validation in 2012.^4,16–20^ While the CMTPedS reliably quantifies overall disability and provides detailed functional profiles, the slow rate of clinical progression in childhood means that short-to medium-term natural history or intervention studies will require large cohorts and/or prolonged follow-up to detect true change. The absence of progression over two to three years should not be interpreted as evidence of treatment efficacy. Rather, it should be viewed as a reflection of the disease’s natural course. This underscores the need to develop more sensitive and responsive biomarkers as surrogate outcome measures, though validating them in such a stable disease context presents challenges.

In Summary, CMTPedS remains a valuable tool for comprehensively assessing childhood CMT. Our study complements existing natural history data yet demonstrating that a significant proportion of pediatric CMT patients, particularly those with mild baseline scores, exhibit clinical stability over two years. These results reinforce the idea that while progression is significant in large, heterogeneous cohorts, it may remain below the threshold of clinical detection in smaller, specific subsets, especially younger children, emphasizing the need for extremely large samples or more sensitive biomarkers in future interventional trials.

## Supplemental material

Supplemental material - Functional disease progression in children with inherited peripheral neuropathies: A prospective cohort study Supplemental material for Functional disease progression in children with inherited peripheral neuropathies: A prospective cohort study by Katharina Vill, Moritz Tacke, Anna König, Jan Senderek, Iris Hannibal, Maggie C. Walter, Beate Schlotter-Weigel, Simone Thiele, Johanna Wagner, Moritz Netzer, Wolfgang Müller-Felber, Elke Hobbiebrunken and Astrid Blaschek in Journal of Neuromuscular Diseases.

Supplemental material - Functional disease progression in children with inherited peripheral neuropathies: A prospective cohort study Supplemental material for Functional disease progression in children with inherited peripheral neuropathies: A prospective cohort study by Katharina Vill, Moritz Tacke, Anna König, Jan Senderek, Iris Hannibal, Maggie C. Walter, Beate Schlotter-Weigel, Simone Thiele, Johanna Wagner, Moritz Netzer, Wolfgang Müller-Felber, Elke Hobbiebrunken and Astrid Blaschek in Journal of Neuromuscular Diseases.

## Data Availability

Detailed data from clinical studies are available from the corresponding author’s institution at the Dr. v. Haunersches Kinderspital, LMU, Munich, Germany, or at the Department of Pediatrics and Adolescent Medicine, University of Göttingen, Germany, upon reasonable request.*

## Appendix

### Abbreviations

CMT: Charcot Marie Tooth.
