# Supplementary material for: Functional disease progression in children with inherited peripheral neuropathies: A prospective cohort study
Source: J Neuromuscul Dis. 2026 Jul 3:22143602261450041. Online ahead of print. doi: 10.1177/22143602261450041 (PMC13438984; doi:10.1177/22143602261450041)
Supplement: Supplementary file 1 [file sj-pdf-1-jnd-10.1177_22143602261450041.pdf]

Supplementary Table 3: Clinical and Genetic Characteristics of the Study Cohort

Patient Number	Year of Birth	Sex	Causative Gene	Genetic Variant	Age at Onset	Symptoms at Onset	Complications	Use of Assistive Devices	Baseline CMTPedS Score
1	2010	m	PMP22	Duplication	9y	Clumsiness, frequent falls, abnormal gait, and muscle pain	—	None	8
2	2011	f	PMP22	Duplication	20mo	Slapping gait and pes cavus	Hip dysplasia	Day and night orthoses	14
3	2006	m	PMP22	Duplication	10y	Difficulty jumping and incipient pes cavus	—	None	14
4	2005	m	PMP22	Duplication	5y	Clumsiness and gait abnormality	—	None	3
5	2011	m	PMP22	Duplication	5y	Slapping gait and frequent falls	—	Insoles	12
6	2003	f	PMP22	Duplication	4y	Unsteady gait, stumbling, clubfoot, and claw toes	Bilateral hallux valgus surgery, EHL transposition, surgical release	Orthopedic shoes	28
7	2007	f	PMP22	Duplication	2y	Pes planovalgus, distal muscle hypotonia, and weakness	Congenital bilateral hip dislocation, hip reconstruction	Insoles	20
8	2004	m	PMP22	Duplication	5y	Difficulty balancing, jumping, and using stairs; talipes equinus	Spondylolisthesis (L5/S1), scoliosis	Insoles, corset	15
9	2006	f	PMP22	Duplication	4y	Difficulty maintaining muscle tension, balance problems, and clumsiness	T1DM, psychiatric comorbidities	AFOs	13
10	2011	f	PMP22	Duplication	3y	Difficulty with stairs, unsteady gait, and difficulty climbing	Bilateral equinocavus foot correction, scoliosis	Orthoses	27
11	2006	f	PMP22	Duplication	3y	Motor uncertainty and pes cavus	Bilateral plantar fasciotomy	AFOs	6
12	2006	m	PMP22	Duplication	21mo	Delayed motor development, frequent falls, and difficulty rising	Severe right hip dysplasia, hip reconstruction, pes planovalgus	Insoles	18
13	2005	m	PMP22	Duplication	2y	Marked external rotation in gait, delayed walking	Bilateral hip surgeries, scoliosis	Insoles, night splint, corset	26
14	2004	f	PMP22	Duplication	2y	Delayed walking and refusal to walk	—	None	24

15	2011	m	PMP22	Duplication	2y	Motor weakness, unsteady gait, and frequent stumbling	—	None	19
16	2008	f	PMP22	Duplication	9y	Slow running and walking-related pain	Peroneal tendon transfer, plantar fasciotomy, clubfoot/pes cavus correction	None	18
17	2009	f	PMP22	Duplication	4y	Gait disorder and pes cavus	—	Insoles, AFOs	12
18	2011	m	PMP22	Duplication	2y	Lower leg pain and abnormal gait	Presumed restless leg syndrome	Insoles	9
19	2001	m	PMP22	Duplication	14y	Progressive pes cavus and muscle cramps	—	Insoles	20
20	2013	f	PMP22	Duplication	8mo	Gross motor stagnation and inability to pull to stand	Flatfoot	Insoles	7
21	2012	f	PMP22	Duplication	3y	High arch of the right foot	—	None	11
22	2008	m	PMP22	Duplication	4y	Delayed gross motor development and difficulty jumping	—	None	14
23	2012	f	PMP22	Duplication	14mo	Increased tiptoe gait	Hip surgery (DVO/Dega)	None	12
24	2005	m	PMP22	Duplication	15mo	Abnormal gait	—	None	11
25	2009	m	PMP22	Duplication	12mo	Walking instability, frequent falls, and foot pain	—	Insoles	8
26	2010	f	PMP22	Duplication	13mo	Increased falling and metatarsus adductus	—	Insoles	7
27	2006	f	PMP22	Duplication	3y	Slow running and difficulty with stairs	—	Insoles	22
28	2003	m	PMP22	Duplication	11mo	Persistent tiptoe gait	—	None	8
29	1999	m	PMP22	Duplication	5y	Asymmetric foot size, pes cavus, and lateral gait emphasis	Metatarsal fracture, ACL tear	Insoles	17
30	2006	f	PMP22	Duplication	6y	Pain and difficulty with prolonged writing	Hip dysplasia, recurrent coxitis	Shoe modifications	20
31	2000	f	PMP22	Duplication	7y	Progressive foot deformity	—	None	27
32	2010	m	MPZ	c.68-1G>A (het.)	13y	Tingling paresthesia and reddish-blue foot discoloration	—	None	6

33	2012	f	MPZ	c.278G>A	2y	Delayed independent walking and knee pain	—	None	15
34	2006	f	MPZ	c.301T>C	18mo	Gross motor delay, slow running, and stairs instability	Foot surgery, thoracic scoliosis, pes cavus	Insoles, corset	25
35	2001	f	MPZ	c.389A>G	2y	Delayed walking and hand tremor	—	None	33
36	2008	f	MPZ	c.161C>T	2y	Frequent falling	—	None	23
37	2004	m	MPZ	c.161C>T	20mo	Gait instability, foot drop, and frequent falls	Pes cavus	None	27
38	2004	f	MFN2	c.1067C>G (het.)	4y	Talipes equinus	Achilles tendon lengthening	AFOs, Nancy Hilton foot pieces	21
39	2006	f	MFN2	c.1090C>T	18mo	Delayed independent standing and walking	—	Power wheelchair, walker, AFOs	43
40	2002	m	MFN2	—	2y	Distal and proximal limb muscle impairment	—	Orthopedic shoes, wheelchair, walker	39
41	1998	m	MFN2	c.310C>T	4y	Great toe extensor weakness and clubfoot deformity	Achilles lengthening, claw toe correction	Active wheelchair, walker, night splints	40
42	2007	f	SH3TC2	c.2860C>T (hom.)	5–6y	Difficulty cycling, climbing, and using stairs; frequent falls	—	Insoles	32
43	2003	m	SH3TC2	c.1894_1897delinsAAA (hom.)	5y	Steppage gait	—	Insoles	23
44	2006	f	SH3TC2	c.2860C>T; c.1757_1758insGCTCC	3y	Delayed tiptoe gait, jumping, and running; calf pain	Hypothyroidism, bilateral plantar fasciotomy, gastrocnemius lengthening	AFOs, night splints	27
45	2001	m	SH3TC2	c.2860C>T; c.1757_1758insGCTCC	2.5y	Impaired running	Spinal fusion, Achilles tendon Z-plasty	None	26
46	2003	m	GJB1	c.283G>A (het.)	7y	Abnormal foot posture	Bilateral CAM impingement	None	10
47	2001	m	GJB1	c.253T>G (hem.)	0y	Pes cavus, talipes equinus, and gait instability	—	Night splints	24
48	2000	m	GJB1	—	0y	Ataxia and progressive pes cavus	Hemiparesis	None	31
49	2002	m	GJB1	c.622G>A	3y	Slow development and difficulty drawing	—	Orthopedic shoes	32

50	2009	m	GDAP1	—	2y	Progressive gait disorder and bilateral claw hands	Bilateral hip dysplasia	Active wheelchair, AFOs, night splints	31
51	2005	f	GDAP1	—	2y	Loss of motor skills and distal limb weakness	Foot surgery (tendon transfer)	DAFO, active wheelchair	37
52	2001	m	GNB4	c.229G>A	18mo	Tiptoe gait and impaired running	Epilepsy, surgical foot correction	Shoe modifications	32
53	2003	m	INF2	c.352_353ACA (het.)	8y	Foot posture changes, abnormal gait, and valgus deformity	—	Night splints	23
54	2007	f	INF2	c.217G>C	21mo	Delayed walking	Achilles tendon lengthening	—	21
55	2002	f	LITAF	c.334G>A (het.)	4y	Frequent falls, difficulty climbing, and poor single-leg jumping	Right hip dysplasia, hip reconstruction	Insoles, orthoses	11
56	2000	m	LMNA	c.892C>T (hom.)	13y	Reduced motor endurance	—	Insoles	20
57	2010	f	NDRG1	c.442C>T (p.Arg148Ter)	3y	Unsteady gait, frequent falls, and leg weakness	—	None	29
58	2008	m	NEFL	c.1186G>A (het.)	8y	Abnormal gait, slow movement, and jumping difficulty	T1DM, Achilles tendon lengthening, plantar release	Night and day orthoses	15
59	2011	f	PMP22	c.97G>T (het.)	6y	Progressive pes cavus	—	None	14
60	2003	f	PMP22	c.215C>T	6mo	Muscle hypotonia and joint hypermobility	Scoliosis	Corset, orthopedic shoes	32
61	2007	m	PRX	c.1390C>T; c.3685C>T	6mo	Proximal weakness and delayed walking	Achilles tenotomy, tibialis posterior transfer	Wheelchair, AFOs, night splints	18
62	2012	f	SACS	c.4744G>A (hom.)	2y	Delayed developmental milestones	—	AFOs, therapy bike	22
63	2007	m	SACS	c.4744G>A (hom.)	12mo	Abnormal standing attempts and delayed walking	—	Insoles, orthoses	31
64	2003	f	Undetermined	—	4y	Abnormal gait, tiptoe gait, and frequent falls	Achilles lengthening, tibialis posterior transfer, peroneal transfer	Insoles, active wheelchair	33
65	2003	f	Undetermined	—	5y	Frequent stumbling	Bilateral foot surgery (tendon transfer, Achilles lengthening)	Orthoses	26
66	2003	m	Undetermined	—	3y	Difficulty with hand tasks	—	Orthoses	12

						and neurogenic pes cavus			
67	2009	m	Undetermined	—	19mo	Frequent stumbling	—	None	10
68	2002	m	Undetermined	—	2y	Delayed walking	Dorsal spinal fusion	Corset, insoles	23
