# Supplementary material for: Functional disease progression in children with inherited peripheral neuropathies: A prospective cohort study
Source: J Neuromuscul Dis. 2026 Jul 3:22143602261450041. Online ahead of print. doi: 10.1177/22143602261450041 (PMC13438984; doi:10.1177/22143602261450041)
Supplement: Supplementary file 2 [file sj-pdf-2-jnd-10.1177_22143602261450041.pdf]

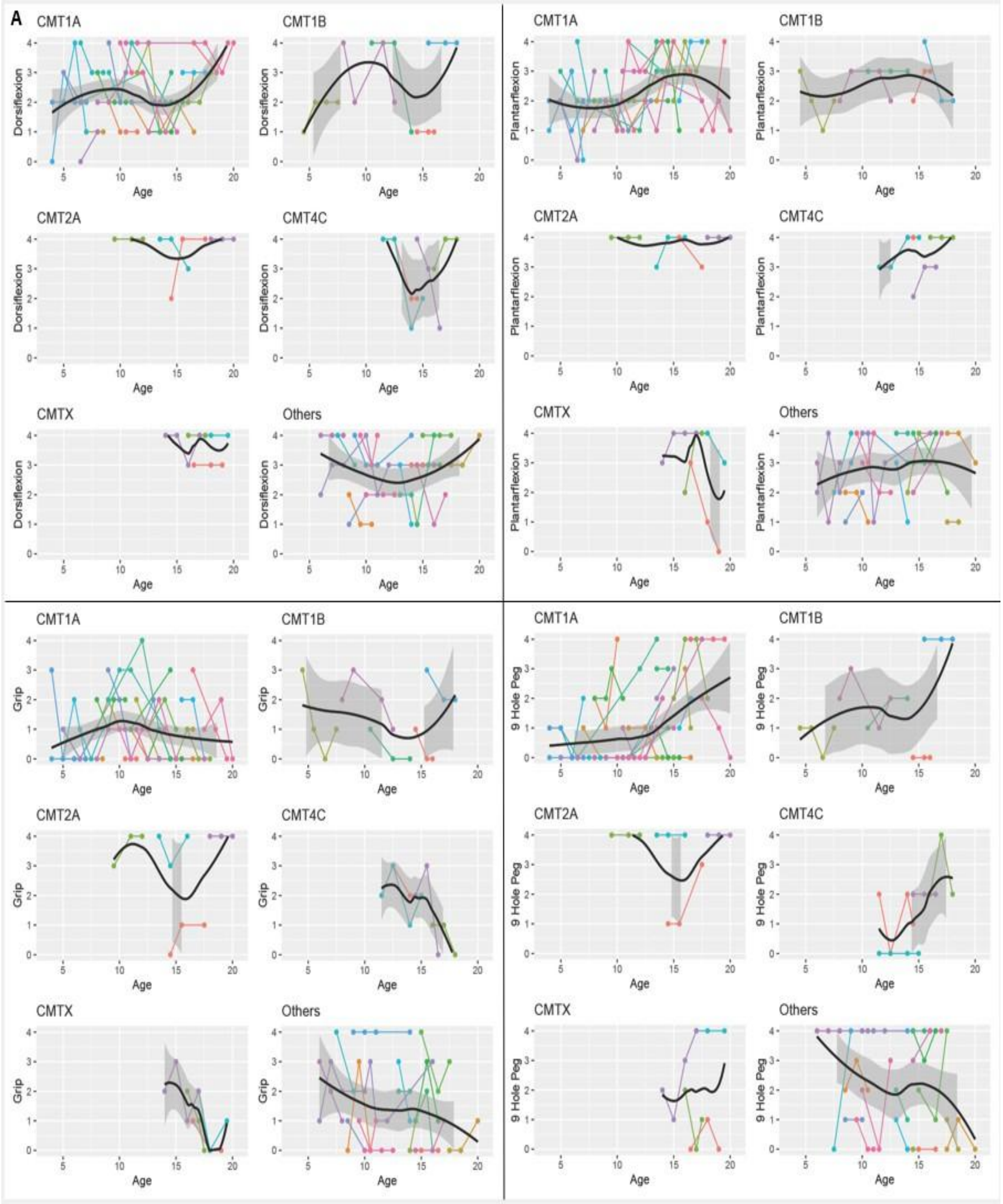

A
CMT1A
CMT1B
CMT2A
CMT4C
CMTX
Others
Dorsiflexion
Plantarflexion
Grip
9 Hole Peg
Age

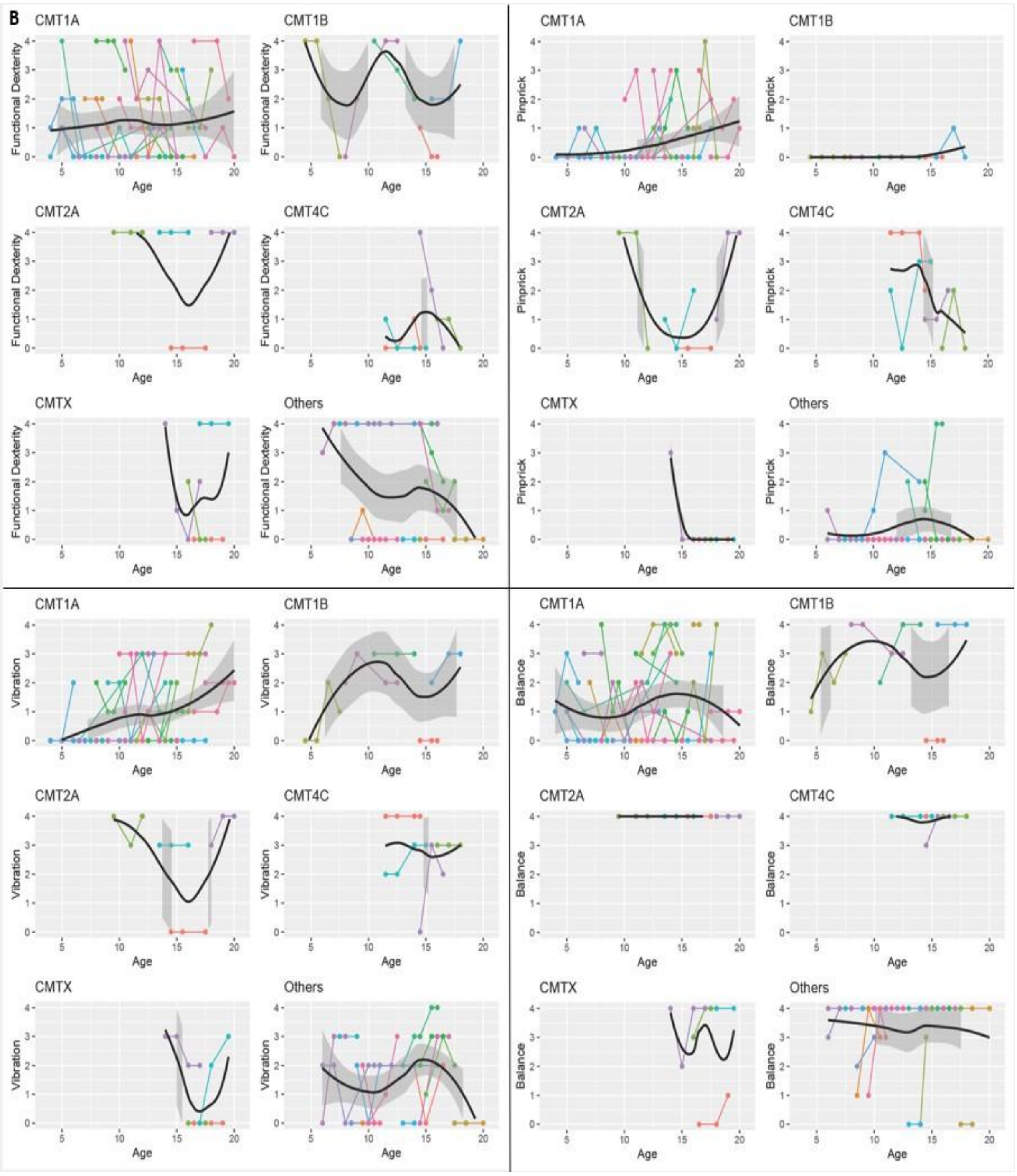
B
CMT1A
CMT1B
CMT2A
CMT4C
CMTX
Others
Functional Dexterity
Pinprick
Vibration
Balance
Age

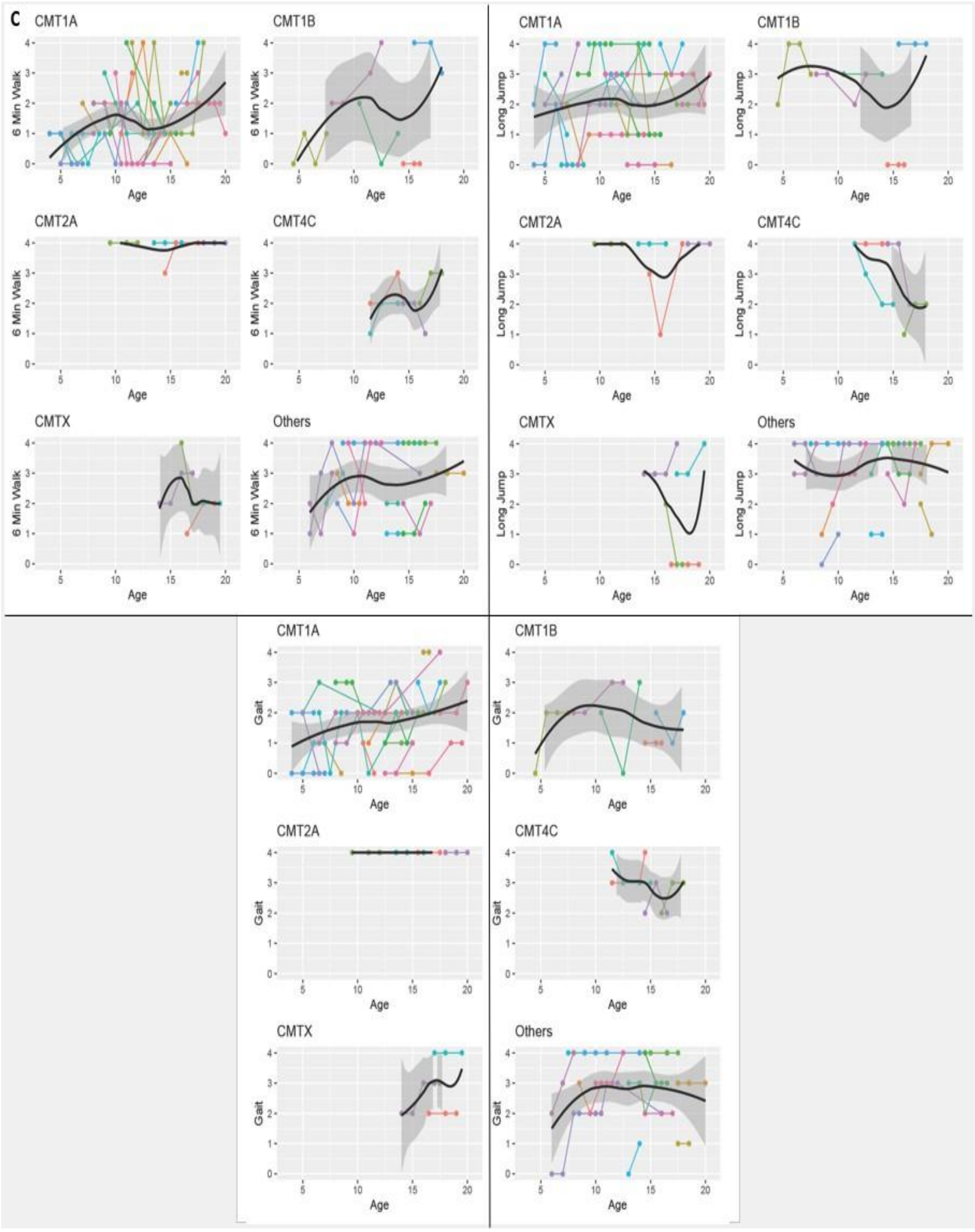
C
CMT1A
CMT1B
CMT2A
CMT4C
CMTX
Others
6 Min Walk
Long Jump
Gait
Age
